# Recovery of inferior alveolar nerve injury after bilateral sagittal split ramus osteotomy (BSSRO): a retrospective study

**DOI:** 10.1186/s40902-016-0068-y

**Published:** 2016-07-05

**Authors:** Chi-Heun Lee, Baek-Soo Lee, Byung-Joon Choi, Jung-Woo Lee, Joo-Young Ohe, Hee-Young Yoo, Yong-Dae Kwon

**Affiliations:** Department of Oral and Maxillofacial Surgery, Graduate School, Kyung Hee University, Seoul, Republic of Korea

**Keywords:** Neurosensory dysfunction, Orthognathic surgery, Vitamin B12

## Abstract

**Background:**

Bilateral sagittal split ramus osteotomy (BSSRO) is the most widely used mandibular surgical technique in orthognathic surgery and is easy to relocate the distal segments, accelerating bone repair by the large surface of bone contact. However, it can cause neurosensory dysfunction (NSD) or sensory loss by injury of the inferior alveolar nerve. The purpose of the present study was to evaluate NSD after BSSRO and modifiers at NSD recovery.

**Methods:**

In this study, NSD characteristics after BSSRO from 2009 to 2014 at the Kyung Hee University Dental Hospital were evaluated. The pattern of sensory recovery over time was also evaluated based on factors such as field of sensory dysfunction, surgical procedure, presence of pre-operative facial asymmetry, and postoperative medications.

**Results:**

Most of the patients had shown NSD immediately after orthognathic surgery. Among the 1192 sides of 596 patients, NSD was observed in 953 sides and 544 patients. Sexual predilection was shown in males (*p* value = 0.0062). In the asymmetric group of 132 patients, NSD was observed in 128 patients (96.97 %). In the symmetric group of 464 patients, NSD was observed in 416 patients (89.45 %); on the other hand, NSD was observed significantly higher in the asymmetric group (*p* = 0.025). NSD-associated factors were analyzed, and vitamin B12 may be beneficial for NSD recovery.

**Conclusions:**

There was a difference between the symmetric group and the asymmetric group in NSD recovery. Vitamin B12 can be regarded as an effective method to nerve recovery. However, a further prospective study is needed.

## Background

Bilateral sagittal split ramus osteotomy (BSSRO) is the most widely used mandibular surgical technique in orthognathic surgery and is easy to relocate the distal segments, accelerating bone repair by the large surface of bone contact. However, it can cause neurosensory dysfunction (NSD) or sensory loss by injury of the inferior alveolar nerve [1].

Dysfunction of the inferior alveolar nerve is indicated by various degrees of numbness on the chin and lower lip area, which is a major postoperative side effect of BSSRO [2]. Postoperative nerve injury is affected by technical factors such as the accuracy of osteotomy; presence of nerve exposure; amount and direction of bony movement; fixation method; surgeon’s skill; anatomy of the mandible; and patient factors such as age, sex, and medical status [3]. Most patients with NSD return to normal after a certain period of recovery time [4].

There has been few controlled studies about nerve injury induced by orthognathic surgery. In this study, we evaluate the recovery of NSD after orthognathic surgery based on several factors, and it can be useful from a clinical perspective.

## Methods

In this retrospective study, 596 patients were evaluated at the Department of Oral and Maxillofacial Surgery, Kyung Hee University Dental Hospital, from 2009 to 2014, who underwent SSRO for the correction of dento-facial deformities. Two hundred eighty-one patients were male and 315 were female, and the mean age of the patients was 24 years old (with standard deviation). All orthognathic surgeries included SSRO; 186 patients underwent SSRO only, and 410 patients underwent SSRO with Le Fort I osteotomy. A Lindemann bur and a reciprocating saw were used for osteotomies, and a chisel, mallet, and separator were used for splitting. The bone fragment was fixed with semi-rigid method by 2.0-mm miniplates and monocortical screws on the planned location. A Hemovac insertion into each operation site and steroid injections for 4 days from surgery were performed to protect postoperative edema. The inter-maxillary fixation with rubber rings was performed on the next day of surgery. During about 5 days hospitalization after the surgery, all the patients were intravenously administered antibiotics, for the prevention of postoperative infection, and vitamin B12 (20mcg/day) for 1 week of hospitalization period.

Postoperative periodic observation and the evaluation sensation were performed at 1 week (immediate postop), 6 months, and 12 months after surgery. The sensation was evaluated independently on both sides through the presence or absence of NSD. A total of 1192 sides from the 596 patients were evaluated. The presence of NSD was asked to the patients such as “Which area felt different on your face?”

The percentage of patients with NSD after SSRO surgery and sensory recovery at the specific time (3, 6, 12 months) were evaluated based on sex, age, and severity of preoperative facial asymmetry.

Preoperative facial asymmetry was evaluated, and the patients were divided into two groups based on the setback or advancement difference of both the right and left bone fragments at the time of operation; the symmetric group had the difference less than 3 mm, and the asymmetric group more than 3 mm. The sensory recovery was instructed as the state of disappearance and absence of NSD. Subjective full sensory recovery of both sides of the face was considered as “normal.” For the statistical analysis, Pearson’s chi-squared test was performed and SPSS 18.0(SPSS Inc, Chicago, IL) was used. And for the analysis of the effect of vitamin B12 administration on NSD, several factors that can affect the NSD are analyzed by using varimax rotation.

## Results

### Incidence of NSD after SSRO surgery

Most of the patients had shown NSD immediately after orthognathic surgery. Among 1192 sides of 596 patients, NSD was observed in 953 sides (80 %) and 544 patients (91 %).

### Effect of sex and age on NSD after SSRO surgery

Among 281 males, 267 males (95 %) had NSD on their face. In the case for females, NSD was observed in 277 (87.9 %) of the 315 patients (Table 1). NSD was observed significantly more in the male group (*p* value = 0.0062).

**Table 1:** Effect of sex and age on NSD

	Gender		Age (year)		
	Male	Female	≤19	20–24	≥25
Normal	14	38	14	24	14
NSD	267	277	99	277	168

There are no statistically significant results in age.

### Effect of preoperative facial asymmetry on NSD after SSRO surgery

**Fig. 1:**
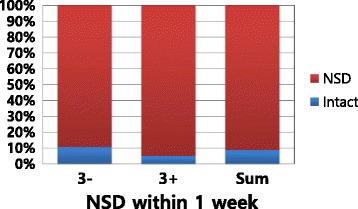
NSD within 1 week

In the asymmetric group of 198 patients, NSD was observed in 188 patients (94.95 %). On the other hand, in the symmetric group of 398 patients, NSD was observed in 356 patients (89.45 %). NSD was observed significantly higher in the asymmetric group (*p* = 0.025) (Table 2 and Fig. 1).

**Table 2:** NSD within 1 week

	Immediate postop.		
Setback difference	NSD	Intact	Total
3−	356	42	398
3+	188	10	198
Total	544	52	596

In the asymmetric group of 198 patents, we compared the incidence of either side of the mandible (interfered side vs. another side). NSD was observed in 155 sides of the interfered side, 128 sides of the another side. It was not statistically significant.

### Sensory recovery of NSD patients

**Fig. 2:**
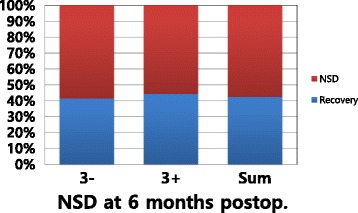
NSD at 6 months postop

**Fig. 3:**
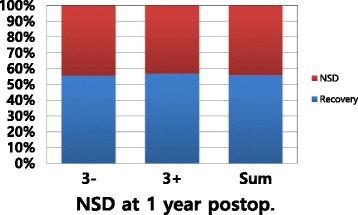
NSD at 1 year postop

Some patients did not visit a clinic during observation period or had missed the evaluation of NSD. They were excluded from the evaluation of the sensory recovery. The patients included in this study were 596 right after surgery, 460 at 6 months, and 338 at 1 year after surgery (Table 3, Fig. 2, Table 4, Fig. 3).

**Table 3:** NSD at 6 months postop

	6 months postop.		
Setback diff.	NSD	Recovery	Total
3−	182	129	311
3+	83	66	149
Total	265	195	460

**Table 4:** NSD at 1 year postop

	1 year postop.		
Setback diff.	NSD	Recovery	Total
3−	102	127	229
3+	47	62	109
Total	149	189	338

The patients who showed NSD immediately after surgery were 544 (91 %) and decreased over time: 265 (57.61 %) at 6 months and 149 (44.08 %) at 1 year after surgery.

In the asymmetric group, NSD was observed in 188 (94.95 %) at immediate post-surgery. They were also decreased over time: 83 (55.70 %) at 6 months and 47 (43.12 %) at 1 year after surgery.

In the symmetric group, NSD was observed in 356 (89.45 %) at immediate post-surgery, 182 (58.52 %) at 6 months, and 102 (44.54 %) at 1 year after surgery.

The difference of NSD between the two groups was observed right after surgery (*p* = 0.025). But, the difference was not any more significant at 6 months (*p* = 0.063) and 1 year (*p* = 0.89).

### The effect of vitamin B12 administration on NSD

The effect of vitamin B12 early administration on NSD was statistically analyzed using varimax rotation. It was significant at 6 months (*p* = 0.0022) and 1 year (*p* = 0.0037) after surgery except at 3 months (Table 5).

**Table 5:** The effect of vit. B12

	The effect of vit. B12		
ANOVA	Recovery at 3 months	Recovery at 6 months	Recovery at 1 year
Vit. B12 adm.	Not significant	5.261 (0.022)	4.386 (0.037)

## Discussion

Inferior alveolar nerve injury has been reported to be common after orthognathic surgery. It may result in the NSD of the lower lip and mentum area. The incidence of NSD has been reported to be approximately 66 to 85 % [3]. In this study, most of the patients who underwent orthognathic surgery presented NSD. However, it decreases over time.

We used an objective neurosensory test such as two-point discrimination and thermal differences. However, the number of study subjects was large; all the 596 patients’ charts have not been recorded perfectly, so subjective evaluation of NSD was not standardized. And the results of the objective and subjective tests of sensory dysfunction are controversial. Cunningham et al. reported an imbalance between the subjective evaluation of sensory dysfunction and the light-touch test, due to the tendency of patients to overestimate sensory dysfunction [5].

Incidence of NSD after SSRO may be influenced by patient age, gender, setback difference, and vitamin B12 administration. Association of NSD with age or gender is controversial. In various studies, age has been found to mediate an effect on the recovery of NSD; particularly in patients older than 40 years, the incidence of NSD was high [6]. But there are studies that report no association of NSD with age or gender [7]. In this study, we observe a significant relation of NSD with gender but not with age.

Although a direct comparison between the interfered side and another side of mandible was not statistically significant, NSD of the interfered side was higher and NSD was observed significantly higher in the asymmetric group rather than the symmetric group. So bony interference caused by advance of setback difference of a bone fragment may exert compressive force on the inferior alveolar nerve, and NSD is expected to occur more frequently on the interfered side. So the preoperative facial asymmetry might be one of the affecting factors in immediate postsurgical NSD.

Regarding the effect of vit. B12, a study reported that vit. B12 and sensory retraining may be beneficial in NSD recovery, but there is also a study against it [8]. Vit. B12 is frequently used for treating peripheral neuropathy, but its efficacy is not clear [9]. In the present study, vit. B12 administration seemed beneficial in the long-term recovery of NSD. Vitamin B plays a vital role in energy metabolism. Nerve tissue may be affected in deficiency states due to its high-energy demand or specific effects of the vitamin. There may also be many factors which have not been considered in the study, so more studies are encouraged.

## Conclusions

In this study, it was found that the incidence of NSD immediately after SSRO surgery was 91 %. However, it decreased over time.

NSD was observed significantly higher in the asymmetric group. It seemed that bony interference caused by advance of setback difference of a bone fragment may exert compressive force on the inferior alveolar nerve, so NSD is expected to occur more frequently on the interfered side. So preoperative facial asymmetry might be one of the affecting factors in immediate postsurgical NSD.

The effect of vitamin B12 early administration on NSD was statistically significant at 6 months and 1 year after surgery. So it can be beneficial in the long-term recovery of NSD.

### Ethics

This study does not disclose any personal information, and the patients are aware that their medical record could be reviewed for research purpose.

## Abbreviations

BSSRO: bilateral sagittal split ramus osteotomy
NSD: neurosensory dysfunction
